# Clinical and Radiographic Parameters for Early Periodontitis Diagnosis: A Comparative Study

**DOI:** 10.3390/dj12120407

**Published:** 2024-12-13

**Authors:** Desy Fidyawati, Sri Lelyati C. Masulili, Hanna Bachtiar Iskandar, Heru Suhartanto, Bramma Kiswanjaya, Xue Li

**Affiliations:** 1 Doctoral Program, Faculty of Dentistry, Universitas Indonesia, Jakarta 10430, Indonesia; desy.fidyawati11@ui.ac.id; 2 Department of Periodontology, Faculty of Dentistry, Universitas Prof. Dr. Moestopo(B), Jakarta 10270, Indonesia; 3 Department of Periodontology, Faculty of Dentistry, Universitas Indonesia, Jakarta 10430, Indonesia; 4 Department of Dento Maxillo Facial Radiology, Faculty of Dentistry, Universitas Indonesia, Jakarta 10430, Indonesia; hanna.bachtiar@ui.ac.id (H.B.I.); bramma.kiswanjaya@ui.ac.id (B.K.); 5 Faculty of Computer Science, Universitas Indonesia, Kota Depok 16424, Indonesia; heru@cs.ui.ac.id; 6 School of Electrical Engineering and Computer Science, University of Queensland, St. Lucia 4072, Australia; xueli@eecs.uq.edu.au

**Keywords:** clinical parameters, radiograph examination, diagnostic accuracy, early periodontitis

## Abstract

**Background/**Objective:**** Early periodontitis diagnosis is challenging due to varying staging and grading systems. While clinical parameters like bleeding on probing (BoP) and pocket depth (PD) are commonly used, periapical radiographs provide valuable information about bone loss and periodontal ligament changes. However, a clear definition of early periodontitis, particularly regarding alveolar bone crest changes, remains elusive. **Methods:** This cross-sectional study involved 21 participants aged 20–30 with clinical signs of periodontitis and radiographic evidence of alveolar bone changes and periodontal ligament widening. Four dentists assessed 40 cases for BoP, 40 for PD, and 40 for periapical radiographs. **Results:** Statistical analysis revealed that the pocket depth measurement was the most significant factor in diagnosing early periodontitis (Fisher’s exact test, *p*-value = 0.000). Additionally, the irregularity of the alveolar crest proved to be a significant marker compared to periodontal ligament width (Fisher’s exact test, *p*-value = 0.000). A Kendall Tau_b test (*p*-value = 0.000, r = 1.000) confirmed pocket depth to be the most influential parameter among the assessed factors. **Conclusions:** While this study highlights the importance of clinical and radiographic assessments in early periodontitis detection, integrating these findings into a definitive diagnosis can be complex. The standardization of diagnostic techniques and the development of advanced radiographic interpretation methods are crucial to improve accuracy. Further research is needed to refine diagnostic criteria and explore additional tests for early periodontitis detection.

## 1. Introduction

The diagnosis of periodontal disease is established after an analysis of the patient’s medical history, clinical picture, and symptom examination through pocket depth examination, loose teeth, radiographic images, blood tests, and biopsy [1]. According to the 2017 World Workshop on the Classification of Periodontal and Peri-Implant Diseases and Conditions, a patient is considered to have periodontal health if their full mouth bleeding on probing score (FMBS) is less than 10%, with probing depths (PPDs) of 3 mm or less and no clinical attachment loss, and patients with gingivitis have bleeding gums in at least 10% of the measured sites (FMBSs) and no tooth loss [2]. A case of periodontitis is defined by an interdental clinical attachment loss of 2 mm or more at two or more non-adjacent teeth or a buccal or oral clinical attachment loss of 3 mm or more with a pocket depth of 3 mm or more for two or more teeth [3,4]. The measurement of the accuracy of the clinical examination parameters is influenced by the accuracy of the intra-rater variables of the fellow observers, which sometimes give different observation results [5]. To analyze these differences, a radiographic examination is needed as an additional examination to obtain the condition of the hard tissue [6].

Radiographic interpretation through 2D radiography for the early detection of periodontal lesions is not easy, as it can only be seen on radiographs after 30–50% of alveolar bone damage has occurred [7]. Hull, William, and Beal (1975) identified three potential criteria for this determination: irregularities in the crestal bone with loss of continuity, the widening of the periodontal ligament, and a distance of more than 3 mm between the alveolar crest and the cementoenamel junction (CEJ) [8]. The dilation of the periodontal ligament is one of the markers of periodontitis. However, in the early symptoms of inflammation, the condition is difficult to detect and observe on radiographic images. The periodontal ligament has an hourglass-like shape, wide on the cervical and apical surfaces, narrow in the middle, and functions as a fulcrum [9,10]. Periodontal ligaments have an average width of 0.15–0.21 mm, which will change with age [10]. Kronfeld (1931) conducted a study on several teeth to determine the width of the periodontal ligament; in the study, the results were obtained on functioning M1 teeth: the width of the periodontal ligament in 1/3 of the cervical area was 0.35 mm, the middle 1/3 was 0.28 mm, and the apical 1/3 was 0.30 mm [11]. Coolidge (1937), in his research, stated that the width of the periodontal ligament depends on the pressure received by the tooth [12]. According to GV Black (1887), normally, the width of the periodontal ligament will decrease with age [13]; according to Bodecker (1926), this is caused by cement deposits at the roots of the teeth that increase with age [14].

Some studies suggest that periapical radiographs are not effective in detecting early alveolar bone damage in periodontal disease. However, in advanced cases of periodontal disease, periapical radiographs tend to reveal more significant bone damage [15,16,17]. The assessment of soft tissue conditions through sulcus depth measurements and hard tissue condition assessments through radiograph examination is one of the markers of periodontal abnormality [18,19]. However, this method often differs between clinicians due to variations in measuring the sulcus depth and differences in radiographic interpretation points of view [20]. The determination of the diagnosis of periodontal disease is carried out based on staging divided into stages I–IV and grading (A-B-C) [4]. The diagnosis of periodontitis is sometimes not simple; this is due to the complex relationship between the predisposing factors involved [21]. Pocket depth, bleeding on probing (BoP), and CAL measurements are still the gold standard for periodontal abnormalities, and radiographs confirm the diagnosis and treatment plan. Still, differences in perception due to probe tip diameter, angulation, probing strength, radiograph interpretation, and intra-examiner differences can result in variations in diagnosis determination [22,23]. Currently, there is no clear agreement on how to define the early stages of periodontitis, particularly when it comes to distinguishing between a normal alveolar bone crest and one that shows signs of damage. Therefore, this diagnostic study aimed to assess the role of clinical examination and lesions on periapical radiographs in the early detection of periodontitis. The null hypothesis posited that the two diagnostic categories were not associated with the onset of early periodontitis.

## 2. Material and Method

The study was conducted in accordance with the Declaration of Helsinki and approved by the Research Ethics Committee of the Faculty of Dentistry, Universitas Indonesia (Protocol No: 070280323, approval date: 27 June 2023), and conducted at the Department of Periodontology, Dental and Oral Hospital, Universitas Indonesia. This study, determining the early diagnosis of periodontitis based on clinical examination and periapical radiographs, was conducted in the context of the research “Analysis of changes in periodontal tissue conditions for early detection of periodontitis using CNN algorithm system on intraoral digital radiography” at the Department of Periodontology, Universitas Indonesia. This is a cross-sectional study; to promote the accuracy of the reporting, the paper was written following the STROBE guidelines [24].

### 2.1. Study Participant

This study collected primary datasets in the form of clinical photographs and periapical radiographs (intraoral digital radiography) that confirmed abnormalities in clinical photos. Four dentists with different levels of clinical experience took part in this investigation; they had more than 20 years of work experience as dentists, especially in the radiograph interpretation for determining the diagnosis of periodontal disease. All of them were lecturers, two for the department of periodontology and another two dentists for the dental radiology department. To avoid bias, two periodontists took clinical data (DF, SLC) alternately; if DF took the data, then SLC observed, and vice versa. Three dental students assisted in this study. They captured clinical photos of BoP and PD using an iPhone 13 camera and directed participants with signs of inflammation to the radiology room for periapical X-rays.

### 2.2. Set of Clinical Examination and Periapical Radiograph

Clinical and radiological information was gathered from study subjects aged 20–30 who qualified for the study, which was performed October–November 2023. All participants gave written consent before undergoing clinical and radiographic examinations. Photos of clinical data, including bleeding on probing and pocket depth measurements, were taken with an iPhone 13 camera, in which the presence or absence of changes in the crest of the alveolar bone and periodontal ligament width were then confirmed. About 40 sites of gingiva were evaluated by each probing pocket depth system and bleeding system using a UNC-15 Probe, and BoP was scored using the Papillary Bleeding Index (PBI) by Saxer and Muhlemnann (1975) [25]. The selected teeth were index teeth, namely 46 44 41 31 34 36 26 24 21 11 14 16, with the criteria of no malposition and no overhanging fillings. All images of periapical radiographs were recorded from the Department of Periodontology at Dental and Oral Hospital, University of Indonesia, utilizing dental X-ray machines with the TRX 708 tube of X-ray intraoral-type CS2200 (Carestream Dental LLC, Cumberland, GA, USA). The exposure time was set at 0.06–0.08 s using a cathode voltage of 60 kV and amperage of 7 mA. Periapical radiographs of the lower and upper incisors, premolars, and molars were selected. Each image needed to capture the full tooth and exhibit at least one irregularity in the alveolar crest or periodontal ligament width. Finally, 40 clinical examination images for BoP, 40 clinical examination images for periodontal ligament width, and 40 periapical radiographs that met the inclusion criteria were identified, and each was assigned a unique identification number.

### 2.3. Diagnostic Standard

**Figure 1:**
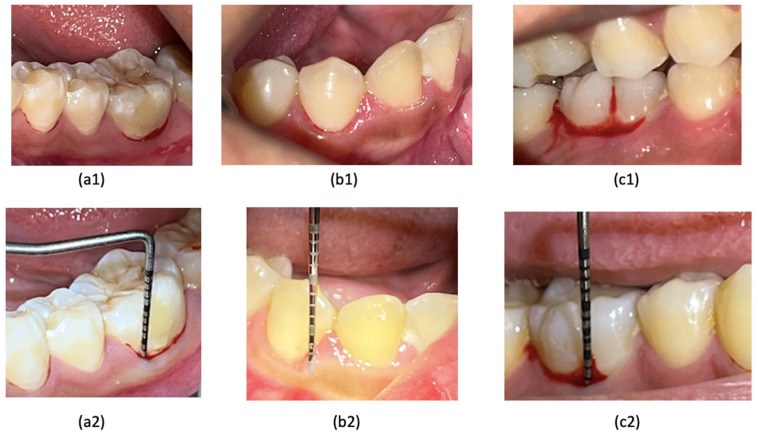
(**a1**–**c1**) Bleeding on probing: (**a1**) tooth 36, (**c1**) tooth 46, BOP with a spreading pattern, (**b1**) tooth 44, BOP with a line pattern (Saxer and Muhlemann (1975) [25], Papillary Bleeding Index). (**a2**–**c2**) The pocket depth measurement for teeth 36, 44, and 46 was found to be less than or equal to 2 mm.

**Figure 2:**
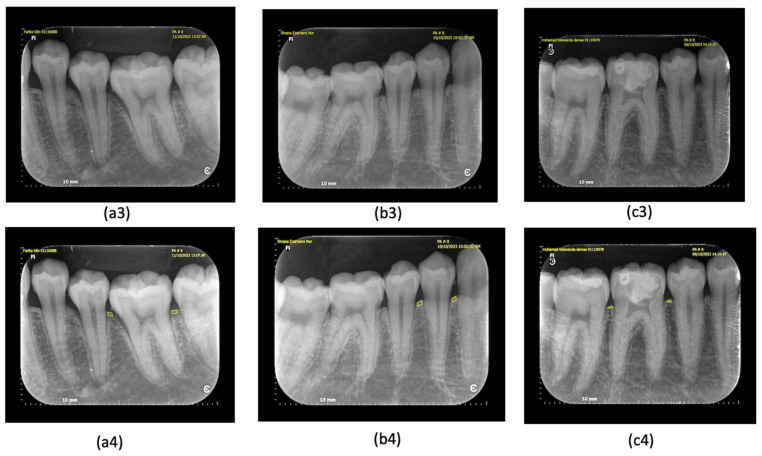
(**a3**–**c3**) Irregularity of alveolar crest and mild bone loss: (**a3**) tooth 36, (**b3**) tooth 44 indicate the presence of marginal irregularities of the alveolar bone crest, (**c3**) tooth 46 indicates a marginal crest that has not changed irregularity. (**a4**–**c4**) Irregularity of alveolar crest and mild bone loss as indicated by the ImageJ application: (**a4**) tooth 36, (**b4**) tooth 44 indicate the presence of marginal irregularities of the alveolar bone crest; (**c4**) tooth 46 indicates a marginal crest that has not changed in irregularity.

**Figure 3:**
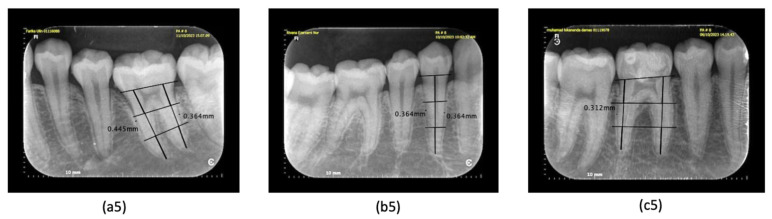
(**a5**–**c5**) To identify early signs of periodontitis, we measured the width of the middle third of the periodontal ligament using ImageJ. (**a5**) Tooth 36 exhibited early symptoms, with a measurement exceeding 0.4 mm. (**b5**,**c5**) In contrast, teeth 44 and 46 displayed a normal periodontal ligament width (0.3–0.4 mm).

All diagnostic standards were defined before the data collection as follows. The criterion of periodontitis was diagnosed according to the new classification of periodontitis [3,26]. When there was bleeding during probing, it may indicate the presence of inflammation in the gingiva (Figure 1) [27], which also comes with a pocket depth >2 mm [3]. In this study, we performed a clinical examination for BoP in advance; if there was bleeding after 10–20 min, probing was carried out to measure the depth of the pocket; a pocket depth of 0–2 mm was still expressed as a normal pocket [1]. On radiographic examination, early detection of periodontitis could be seen in the irregularity of the alveolar crest in the proximal area, as well as the presence of a non-pointed image of the alveolar crest in the proximal area of the anterior teeth (Figure 2) [28]. The marginal cortical crest of the alveolar bone that was irregular was marked, the shape of the cortical bone crest was irregular (unsmooth), or there was an indentation; there is a picture of a radiolucency zone at the cortical bone crest [10,15]. Another way to detect early periodontitis is to measure the thickness of the periodontal ligament (Figure 3). This measurement is taken in the middle third of the ligament [9], at the homeostatic phase on anterior teeth typically measuring 0.2 mm or posterior teeth measuring 0.3 to 0.4 mm [11,12]. Gingivitis is a precursor of periodontitis and can be reversed if diagnosed early.

Bleeding on probing was recorded when there was spontaneous bleeding from the gingival when probing at an optimal pressure of 0.25 N (20–25 g); the probe’s tip may damage vessels within the second layer of the subepithelial plexus and the epithelium of the pocket wall, leading to gingival bleeding. This bleeding can serve as a diagnostic indicator of periodontal tissue inflammation (Figure 1a1–c1) [29]. The depth of the periodontal pockets around teeth 36, 44, and 46 was measured to be 2 mm or shallower (Figure 1a2–c2), indicating a normal periodontal pocket [3]. In the early stages of periodontitis, the alveolar crest becomes blunted, and the height of the alveolar bone decreases in the anterior region. The posterior region may also show a loss of the normally sharp angle between the lamina dura and the alveolar crest (Figure 2a3–c3) [10]. The irregularity of the alveolar bone crest, analyzed using the ImageJ application, reveals an uneven cortical bone crest shape (not smooth) or indentations, along with a description of a radiolucent area at the cortical bone crest (Figure 2a4–c4) [30]. Meanwhile, periodontal ligament width measurements using ImageJ indicated an initial periodontitis lesion, evidenced by a dilation of the middle third of the periodontal ligament exceeding 0.3–0.4 mm (Figure 3a5–c5) [11].

### 2.4. Consensus Decision (Reference Standard)

After the screening assessments, all four dentists re-examined the clinical and radiographic images to achieve a consensus. A yes/no decision (0 or 1) was made for the chosen criteria related to the clinical picture (BOP and pocket depth) and the periapical image (the irregularity of the marginal crest of the alveolar bone and periodontal ligament width). Decision (0) means healthy, and (1) means early periodontitis. If any participant had a differing result, the team members re-evaluated and discussed it until they reached an agreement, which was then established as a reference.

### 2.5. Data Management and Statistical Analysis

An Excel spreadsheet (Excel 2019, Microsoft Excel for Mac, Redmond, WA, USA) was utilized to gather results from the four dentists, the two assessments (*n* = 2), and the reference standard. The spreadsheet was verified for accuracy before analysis. Descriptive and exploratory data analyses were conducted using Excel and SPSS (SPSS Statistics 26, 2020, IBM Corporation, Armonk, NY, USA). Kendall’s tau correlation was employed to analyze the linear association between two or more categorical variables; in this study, we aimed to observe the correlation between clinical examination (BOP and pocket depth) and radiography examination (the irregularity of the alveolar crest and periodontal ligament width). Since the data were categorical, a normality test was not required. The r-value, ranging from 0.00 to 1.00, represents the correlation coefficient. A value between 0.00 and 0.25 indicated a weak correlation, 0.26 to 0.50 signified a moderate correlation, 0.51 to 0.75 suggested a strong correlation, and 0.76 to 1.00 denoted a very strong correlation. The analysis considered the diagnostic decision based on two examinations, a clinical examination and periapical radiograph, to conduct a more detailed analysis of the variables; further comparative tests were implemented between the two groups, and a categorical comparative analysis was conducted using Fisher’s exact test to assess the relationship between clinical and radiographic parameters in the early diagnosis of periodontitis. This analysis aimed to identify the key parameters that contribute to accurate diagnosis.

## 3. Result

**Figure 4:**
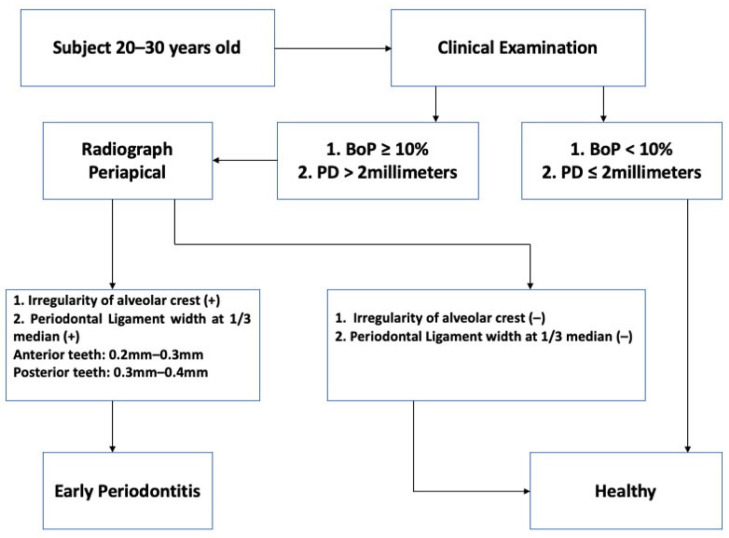
The flowchart shows the sample recruitment process.

The tables below provide detailed data for all dentists involved in the study. We collected primary data from 21 qualified participants, including 40 clinical assessments of bleeding on probing (BOP) and 40 pocket depth (PD) assessments. The same pattern was observed in 40 periapical images, which depicted irregular and dilated periodontal ligaments surrounding the index teeth. The flowchart below outlines the steps involved in selecting and enrolling samples in the study (Figure 4).

Table 1 provides demographic data detailing the number of healthy teeth and those with early periodontitis, with two parameters of clinical examination (BoP and PD) and another two parameters of periapical radiograph examination (irregularity of alveolar crest and periodontal ligament width). A clinical examination using BoP identified early periodontitis in 97% (39 samples) of cases. However, pocket depth measurements detected it in only 7.5% of cases (three samples). A radiographic examination, using both alveolar crest irregularity and periodontal ligament dilation, identified early periodontitis in 60% (24 samples) of cases for both parameters.

Table 2, Table 3, Table 4 and Table 5 analyze the association between clinical (BoP, PD) and radiographic (alveolar crest irregularity and periodontal ligament width) parameters in differentiating between healthy and early periodontal conditions. While BoP, alveolar crest irregularity, and periodontal ligament dilation were not significantly associated with early periodontitis (*p*-value > 0.05), PD was found to be statistically significant (*p*-value < 0.05) (Table 3).

To determine the most effective combination of parameters for initial diagnosis, Table 6, Table 7, Table 8, Table 9 and Table 10 compare these parameters pairwise between clinical parameters and radiographic parameters. Table 6 compares healthy/BoP(–) and early periodontitis/ BoP(+) samples based on the clinical parameter of bleeding on probing (BoP). Of the samples identified as early periodontitis by BoP(+), 61.5% also exhibited alveolar crest irregularity on the radiographic examination. In Table 7, BoP was compared to another radiographic parameter, periodontal ligament width. Similarly to the previous comparison, 61.5% of samples with BoP(+) also showed periodontal ligament dilation. Table 8 compares the clinical parameter of pocket depth (PD) with alveolar crest irregularity. Only 66.7% of the samples diagnosed with early periodontitis based on PD > 2 mm also exhibited alveolar crest irregularity on the radiographs. Table 9 compares PD with periodontal ligament dilation. Similarly, 66.7% of samples with PD > 2 mm and early periodontitis also showed periodontal ligament dilation on the radiographs. The comparison between the radiographic parameters (irregularity and dilation) showed the highest statistical significance (Table 10, *p*-value = 0.000). Table 10 demonstrates that each of the 24 samples (61.5%) displaying periodontal ligament dilation also presented with alveolar crest irregularity. In this study, alveolar crest irregularity was found to be a more significant factor for determining early periodontitis than periodontal ligament dilation.

To identify the most influential parameter in detecting early periodontitis, the Kendall Tau_b test was conducted. Table 11 reveals that pocket depth is the most influential clinical parameter, with a strong positive correlation (r = 1.000). Among other parameters, pocket depth can serve as a reliable indicator for early periodontitis detection (normally ≤ 2 mm) [3].

## 4. Discussion

Gingival inflammation serves as an early indicator of periodontal disease. If left untreated, gingivitis can progress to periodontitis, a more advanced stage characterized by the destruction of the gingival tissue, bone, and periodontal ligament. This destructive process results in the formation of deep periodontal pockets, ultimately increasing the risk of tooth loss [31]. The number of patients diagnosed with gingivitis, as per the 2018 classification using BOP% [23], was sixty-six percent lower compared to those diagnosed through a visual assessment and BOP% [32]. These results confirm that visual indicators of inflammation, such as alterations in gingival color and volume, can be found in cases of healthy gingiva where BOP% is less than 10% [33]. While the 2018 classification for gingivitis is easy to use, the results suggest that it can lead to significant differences in diagnoses and treatment plans among clinicians when they adopt this new system [33]. Table 1 illustrates the samples of each parameter (*n* = 40) based on clinical examinations and radiography examination, which stated that 39 samples (97%) of parameter BoP indicated early periodontitis, and only three samples of PD indicated early periodontitis. In this table, it is also explained that based on the periapical radiography examination, through irregular alveolar crest and periodontal ligament width, 24 samples (60%) were diagnosed with early periodontitis. A recent study evaluating practitioners’ grasp of the 2018 PD classification system found that diagnosing gingivitis was the most difficult. It reported that 65–95% of periodontal specialists and general practitioners misclassified a healthy gingiva case as biofilm-induced gingivitis [27]. The early manifestations of periodontitis are clinically recognizable; furthermore, staging based on radiographic bone loss is important since the extent of alveolar bone changes can be visualized more accurately [34,35]. Table 2, Table 3, Table 4 and Table 5 analyze the relationship between various clinical and radiographic parameters in determining the diagnosis of early periodontitis. Among these parameters, pocket depth measurement demonstrated the highest statistical significance (*p*-value = 0.000) in diagnosing early periodontitis, surpassing the significance of other parameters such as bleeding on probing (BoP), alveolar crest irregularity, and periodontal ligament dilation. Based on the 2017 classification of periodontal diseases, there is a general association between bleeding on probing (BoP), pocket depth (PD), alveolar crest irregularity, and periodontal ligament dilation. If the percentage of sites with BoP increases significantly (more than 10%) without evidence of radiographic bone loss (RBL) or clinical attachment loss (CAL), the condition is typically classified as gingivitis [4]. However, for most patients, their BOP% did not meet the 2018 diagnostic criteria to classify as a case of gingivitis with intact periodontium [33]. A higher percentage of BoP often correlates with deeper periodontal pockets. Periodontitis develops when this pocket deepening is accompanied by damage to the alveolar bone, which is manifested at the earliest radiographically for alveolar crest irregularity and periodontal ligament dilation [8]. The accurate diagnosis of periodontal disease requires both clinical and radiographic examinations. An initial clinical examination involves assessing factors like bleeding on probing, pocket depth, and attachment loss. Periapical radiography is commonly used to confirm these findings due to its affordability, accessibility, and low radiation exposure [36]. Table 6, Table 7, Table 8, Table 9 and Table 10 provide a detailed analysis of the interrelationship between four key parameters in the initial diagnosis of periodontitis. The study reveals that radiographic parameters, particularly alveolar crest irregularity and periodontal ligament dilation, play a more significant role in accurate diagnosis compared to clinical parameters. This increased influence of radiographic parameters can be attributed to their ability to provide a visual representation of underlying bone and tissue changes that may not be apparent during a standard clinical examination. Table 10 further emphasizes this point, demonstrating that samples exhibiting periodontal ligament dilation consistently present with alveolar crest irregularity. This combination of radiographic findings can serve as a valuable diagnostic tool for the early detection of periodontitis. This study, as presented in Table 11, indicates that pocket depth is the most influential clinical parameter, with a very strong positive correlation (r = 1.000). This strong correlation implies that pocket depth can serve as a reliable indicator for the early detection of periodontitis. Gingival bleeding is an early indicator of inflammation and was first included in the clinical periodontal index in 1958 [37]. The 2017 World Workshop introduced the concept of “pristine periodontal health,” which signifies the absence of clinical inflammation. A periodontal probe is a primary diagnostic tool, and its accuracy is influenced by factors such as probe design, probing force, and probe position. Clinical signs detected through probing include bleeding, pocket depth, and attachment loss. The 2017 World Workshop emphasizes that probing force directly impacts the prevalence of bleeding on probing [38]. To the best of our knowledge, this is the first study to analyze the correlation between clinical examinations (BoP and PD) and radiograph findings (irregularity of alveolar crest and periodontal ligament width) for the early detection of periodontitis according to the 2018 Classification. Periapical radiographs provide a two-dimensional view, which can sometimes distort the true three-dimensional relationships between teeth and bone structures, and overlapping anatomical structures can obscure or complicate the interpretation [10]. Clinical examinations, on the other hand, can be somewhat subjective, with different clinicians potentially interpreting the findings differently based on their experience and technique [1]. The study by Milgrom et al. revealed that clinical knowledge and technical skills in dental radiography impacted the frequency of diagnostic errors. Factors linked to clinical knowledge included inadequate interpretation and the improper use of radiographs [39].

Clinical examinations are inherently subjective. In bleeding-on-probing (BoP) assessments, the results can be influenced by factors such as the use of periodontal probes and the specific technique used for placing these probes in the gingival sulcus, which can impact clinical judgments; similarly, detecting the marginal irregularity of the alveolar bone crest in periapical radiographs demands a high level of clinical skill and experience, making it somewhat subjective at times. In the early stages of periodontitis, periapical radiographs cannot provide a complete picture of the disease. This is because radiographic changes, such as bone loss, only become visible after the damage of 30–50% of the alveolar bone [7]. Additionally, periapical radiographs do not provide information on clinical parameters like pocket depth and tooth mobility [40]. The samples used were also limited, so more samples are needed to see the compatibility between the clinical picture and periapical radiograph. The strength of this study is that the collection of clinical examination data, the analysis of clinical examination, and the radiographic examination were carried out by periodontists and dental radiology specialists. In this context, it is important to note that the clinical experience of the dentists significantly impacted the study’s outcomes. Experienced dentists were better at identifying pathologies during clinical examinations and on periapical radiographs, highlighting the need for the early detection of periodontitis.

## 5. Conclusions

In conclusion, combining clinical and radiographic evidence requires careful analysis to prevent misdiagnosis. Radiographic bone loss may not always match clinical findings due to anatomical differences or disease progression. Integrating clinical and radiographic data for accurate diagnosis can be complex, especially in challenging cases. More experienced dentists tend to make more reliable assessments. Future research should focus on developing additional tests to improve the accuracy of radiographic interpretation.

Future efforts should aim at establishing supplementary examinations to enhance the accuracy of radiographic interpretation.

## Figures and Tables

**Table 1:** Demographic data describing the number of healthy teeth and teeth with early periodontitis based on clinical examination and radiograph examination.

Examination	Parameters	Healthy (*n*)	Early Periodontitis (*n*)	Total (*n*)
Clinical Examination	Bleeding on probing (BoP)	1 (2.5%)	39 (97.5%)	40
	Pocket depth (PD)	37 (92.5%)	3 (7.5%)	40
Periapical Radiograph Examination	Irregularity of alveolar crest	16 (40%)	24 (60%)	40
	Periodontal ligament width (PDL)	16 (40%)	24 (60%)	40

**Table 2:** Comparative test of bleeding on probing to determine the diagnosis of early periodontitis.

Diagnose Decision	Bleeding on Probing (BoP)		*p*-Value
	No BoP	There is BoP	
Healthy (*n* = 37)	1 (2.7%)	36 (97.3%)	1.000 *
Early Periodontitis (*n* = 3)	0 (0.0%)	3 (100%)	

* Fisher’s exact test.

**Table 3:** Comparative test of pocket depth to determine the diagnosis of early periodontitis.

Diagnose Decision	Pocket Depth (PD)		*p*-Value
	No PD	There is PD	
Healthy (*n* = 37)	37 (100%)	0 (0.0%)	0.000 *
Early Periodontitis (*n* = 3)	0 (0.0%)	3 (100%)	

* Fisher’s exact test.

**Table 4:** Comparative test of irregularity of alveolar crest, to determine the diagnosis of early periodontitis.

Diagnose Decision	Irregularity of Alveolar Crest		*p*-Value
	No Irregularity	There Is Irregularity	
Healthy (*n* = 37)	15 (40.5%)	22 (59.5%)	1.000 *
Early Periodontitis (*n* = 3)	1 (33.3%)	2 (66.7%)	

* Fisher’s exact test.

**Table 5:** Comparative test of periodontal ligament width to determine the diagnosis of early periodontitis.

Diagnose Decision	Periodontal Ligament Width (PLW)		*p*-Value
	No PLW	There Is PLW	
Healthy (*n* = 37)	15 (40.5%)	22 (59.5%)	1.000 *
Early Periodontitis (*n* = 3)	1 (33.3%)	2 (66.7%)	

* Fisher’s exact test.

**Table 6:** Comparative test of pocket depth for healthy and early periodontitis cases with BoP as a clinical parameter.

Bleeding on Probing (BoP)	Irregularity of Alveolar Crest		*p*-Value
	No Irregularity	There Is Irregularity	
Healthy/BoP (–) (*n* = 1)	1 (100%)	0 (0.0%)	0.400 *
Early Periodontitis/ BoP (+) (*n* = 39)	15 (38.5%)	24 (61.5%)	

* Fisher’s exact test.

**Table 7:** Comparative test of periodontal ligament width for healthy and early periodontitis cases with BoP as a clinical parameter.

Bleeding on Probing = (BoP)	Periodontal Ligament Width (PLW)		*p*-Value
	No PLW	There Is PLW	
Healthy/BoP (–) (*n* = 1)	1 (100%)	0 (0.0%)	0.400 *
Early Periodontitis/ BoP (+) (*n* = 39)	15 (38.5%)	24 (61.5%)	

* Fisher’s exact test.

**Table 8:** Comparative test of irregularity of alveolar crest for healthy and early periodontitis cases with pocket depth as a clinical parameter.

Pocket Depth	Irregularity of Alveolar Crest		*p*-Value
	No Irregularity	There Is Irregularity	
Healthy/PD (–) (*n* = 37)	15 (40.5%)	22 (59.5%)	1.000 *
Early Periodontitis/ PD (+) (*n* = 3)	1 (33.3%)	2 (66.7%)	

* Fisher’s exact test.

**Table 9:** Comparative test of periodontal ligament width for healthy and early periodontitis cases with pocket depth as a clinical parameter.

Pocket Depth (PD)	Periodontal Ligament Width (PLW)		*p*-Value
	No PLW	There Is PLW	
Healthy/PD (–) (*n* = 37)	15 (40.5%)	22 (59.5%)	1.000 *
Early Periodontitis/ PD (+) (i = 3)	1 (33.3%)	2 (66.7%)	

* Fisher’s exact test.

**Table 10:** Comparative test of irregularity of alveolar crest for healthy and early periodontitis cases with periodontal ligament width as a radiograph parameter.

Periodontal Ligament Width (PLW)	Irregularity of Alveolar Crest		*p*-Value
	No Irregularity	There Is Irregularity	
Healthy/PLW (–) (*n* = 16)	16 (100%)	0 (0.0%)	0.000 *
Early Periodontitis/ PLW (+) (*n* = 24)	0 (0.0%)	24 (100%)	

* Fisher’s exact test.

**Table 11:** Correlation of determining early periodontitis based on clinical examination and radiograph examination.

Parameter	Diagnose Decision	
	Correlation Coefficient (r)	Sig (2-Tailed)
Bleeding on probing	0.95	0.553
Pocket depth	1.000	0.000 *
Irregularity	0.39	0.809
Periodontal ligament width	0.39	0.809

* Significant correlation (Kendall Tau_b test).

## Data Availability

The datasets generated and analyzed during the current study are obtainable from the corresponding author upon reasonable request.
